# Clinical Course of Four Patients with Mucormycosis Managed Predominantly with Ayurveda-Based Interventions: A Case Series

**DOI:** 10.3390/jof12100781

**Published:** 2026-10-19

**Authors:** Rajnikant Patel, Skanthesh Lakshmanan, Vinayak Padikkal, Maël Voegeli, Keyur Mandaliya

**Affiliations:** 1 Agnivesh Ayurved Hospital, Surat 395010, India; kachhadiyarajnikant@gmail.com; 2 Ayurveda Division, Centre for Advanced Studies and Integrated Research in Ayurveda, Thrissur 680020, India; 3 Clinical Research and Ethics Division, Centre for Advanced Studies and Integrated Research in Ayurveda, Thrissur 680020, India; 4 Public Health Division, Centre for Advanced Studies and Integrated Research in Ayurveda, Thrissur 680020, India; 5 Ayu Emergency Care Research, ICU India, Coimbatore 641045, India; 6 Prismaa Oncoimaging Surat, Surat 395002, India

**Keywords:** mucormycosis, ayurveda, case series, emergency care, complex disease management

## Abstract

Mucormycosis is a rapidly progressive angioinvasive fungal infection caused by Mucorales, predominantly affecting immunocompromised individuals and associated with substantial morbidity and mortality. Despite advances in standard care involving surgical debridement and systemic antifungal therapy, outcomes may remain suboptimal because of recurrence, treatment-related toxicity, including nephrotoxicity associated with amphotericin B, and substantial economic burden. These challenges justify continued investigation of adjunctive therapies and other approaches that may potentially improve outcomes or reduce treatment burden, while established antifungal therapy and surgical management remain the cornerstone of care. Ayurveda, a traditional system of medicine in India, employs comprehensive therapeutic strategies that may include oral formulations, local interventions, dietary regulation, and lifestyle modifications. Ayurveda has been utilised by patients with mucormycosis; however, its evidence base remains limited and largely restricted to adjunctive or isolated case reports. This paper presents a descriptive case series of four patients diagnosed with mucormycosis who were managed predominantly with Ayurveda-based interventions. Three patients opted to receive stand-alone Ayurveda-based treatment after declining recommended standard biomedical treatment. All patients had complex comorbidities, including diabetes mellitus, hypertension, and renal impairment. Serial follow-up imaging documented radiological changes interpreted by the reporting radiologists as improvement in three patients, with no documented recurrence during the available follow-up periods. One patient with suboptimal treatment adherence showed persistent radiological abnormalities at the three-month assessment, although survival was maintained for approximately one year following diagnosis. No treatment-related adverse events were documented in the available follow-up records; one patient with pre-existing renal impairment showed an increase in serum creatinine from 1.48 to 3.3 mg/dL during follow-up. These observations are descriptive and derive from an uncontrolled case series with non-standardised follow-up and outcome assessment. They do not establish treatment effectiveness, safety, non-inferiority, or suitability for clinical application and should not be interpreted as evidence supporting replacement of current standard-of-care management. Rather, these observations advance ongoing hypothesis generation and provide conceptual and methodological insights for the development of future clinical research protocols evaluating predominantly Ayurveda-based interventions for mucormycosis care.

## 1. Introduction

Mucormycosis is a rare, rapidly progressive, angioinvasive fungal infection caused by filamentous fungi belonging to the order Mucorales [1]. The disease predominantly affects individuals with impaired host immunity, particularly those with uncontrolled diabetes mellitus, haematological malignancies, recipients of solid-organ or haematopoietic stem cell transplantation, and patients receiving prolonged immunosuppressive therapy [2]. Following tissue invasion, the fungi exhibit a marked propensity for vascular infiltration, leading to thrombosis, tissue ischaemia, and extensive necrosis [3]. Infection typically originates within the nasal cavity and paranasal sinuses and may rapidly extend to the orbit, cranial nerves, skull base, and intracranial structures [1]. Among the various clinical manifestations, rhino-orbito-cerebral mucormycosis (ROCM) is the most frequently encountered presentation and remains associated with mortality rates ranging from approximately 30% to 50% [4].

Beyond its substantial mortality, mucormycosis imposes considerable long-term morbidity. Survivors frequently experience irreversible visual impairment, cranial neuropathies, maxillofacial defects, facial disfigurement, and persistent functional limitations affecting mastication, speech, swallowing, and nasal breathing, all of which profoundly compromise health-related quality of life [5]. Consequently, mucormycosis constitutes a medical emergency that demands timely diagnosis and coordinated multidisciplinary management [1].

Contemporary management relies on a multimodal therapeutic strategy comprising early diagnosis, prompt reversal of underlying predisposing factors whenever feasible, initiation of systemic antifungal therapy with high-dose liposomal amphotericin B as the preferred first-line agent, aggressive surgical debridement of necrotic tissue, and step-down therapy with oral triazoles such as posaconazole or isavuconazole when clinically indicated [6]. The implementation of these evidence-based interventions through multidisciplinary care has substantially improved survival compared with historical outcomes and remains the cornerstone of mucormycosis management [7].

Nevertheless, important clinical challenges persist despite these therapeutic advances. Overall case-fatality rates remain at approximately 40–50%, varying according to the anatomical site of infection, disease extent, underlying comorbidities, and timeliness of intervention [1]. Furthermore, radical surgical procedures frequently result in permanent anatomical and functional deficits, while prolonged antifungal therapy may lead to nephrotoxicity, hepatotoxicity, electrolyte disturbances, infusion-related adverse effects, and considerable financial burden [8,9]. Persistent or recurrent disease also continues to occur despite apparently appropriate medical and surgical management, particularly among patients with delayed presentation or persistent immunosuppression [10]. Collectively, these challenges underscore the need to investigate additional evidence-based therapeutic strategies aimed at preserving tissue integrity, improving functional recovery, reducing treatment-related morbidity, and enhancing patient-centred outcomes, while recognising that currently established systemic antifungal therapy and surgical management remain the standard of care for mucormycosis. Ayurveda is a traditional system of medicine originating in the Indian subcontinent that conceptualises health and disease through a holistic framework. Its therapeutic approach is tailored to the clinical presentation and individual characteristics of the patient and may incorporate internal medicinal formulations, local or external therapies, dietary regulation, and lifestyle modifications [11]. It continues to play an important role in healthcare delivery across the country [12]. The COVID-19 pandemic has caused a significant clinical burden for the management of mucormycosis [13].

During the COVID-19 pandemic, disruptions in healthcare delivery, restricted access to conventional medical services, and increased public interest in complementary healthcare were associated with greater utilisation of Ayurveda and other traditional medical systems [14]. This pattern of healthcare-seeking extended to patients with COVID-19-associated complications, including mucormycosis who sought Ayurveda either as a primary therapeutic approach or as an adjunct to conventional management [15].

Despite its widespread utilisation, robust clinical evidence supporting Ayurveda in the management of mucormycosis remains limited [16]. This evidence gap presents two important challenges. On the one hand, potentially promising therapeutic approaches cannot be rigorously evaluated or appropriately integrated into evidence-informed clinical practice [17]. On the other hand, the absence of robust evidence creates uncertainty regarding efficacy and safety, thereby limiting informed clinical decision-making and potentially affecting patient safety as well [11]. Accordingly, systematic evaluation of Ayurveda-based interventions is essential to establish an objective and scientifically robust evidence base [18].

Generating high-quality evidence in mucormycosis presents substantial methodological challenges. The rarity of the disease, rapid clinical progression, marked heterogeneity in patient characteristics, and ethical constraints surrounding randomisation in life-threatening conditions limit the feasibility of adequately powered randomised controlled trials [19]. Consequently, well-conducted observational studies, rigorously documented case series, and other forms of real-world clinical evidence assume an important role in characterizing therapeutic responses, generating hypotheses, and informing future clinical research and guideline development [1].

Experimental investigations have demonstrated antifungal, anti-inflammatory, and immunomodulatory properties of several Ayurvedic medicinal plants and formulations against clinically relevant fungal pathogens, providing preliminary experimental rationale [20]. However, the available clinical literature on Ayurveda in mucormycosis remains limited to non-randomised studies evaluating Ayurveda as an adjunct to conventional treatment, together with a single published case report describing successful stand-alone Ayurveda management [15,21]. Consequently, important evidence gaps persist regarding the evaluation of predominantly Ayurveda-based management, particularly with respect to objective clinical and radiological outcomes, safety, and long-term follow-up.

To our knowledge, this is one of the first case series describing predominantly Ayurveda-based management of mucormycosis, with comprehensive documentation of therapeutic strategies, serial clinical and radiological outcomes, and long-term follow-up. As one of the first studies of its kind, this work contributes to the emerging evidence base by providing preliminary real-world clinical evidence to inform future hypothesis-driven research on the possible relevance of Ayurveda in mucormycosis management.

## 2. Diagnostic Framework

The diagnostic framework adapted in this case series uses a pragmatic multimodal clinical framework integrating compatible clinical features, direct microscopy and fungal culture where available, histopathological demonstration of fungal invasion, and supportive radiological findings. Diagnostic certainty was determined case by case according to the available evidence rather than by a single microbiological criterion. Where more than one fungal organism or morphologically distinct fungal element was identified, the possibility of mixed fungal rhinosinusitis was documented.

## 3. Outcome Assessment

Outcome assessment was based on adapted measures derived from routine clinical documentation, including clinical observations and radiological reports. As this was a case series based on real-world clinical practice, follow-up intervals and symptom assessments were not standardised. Radiological findings were obtained from routine reports and were not independently reviewed by blinded assessors. Accordingly, the reported outcomes should be interpreted as descriptive clinical observations rather than standardised or independently adjudicated outcome measures.

### 3.1. Case 1

A 65-year-old male from western India with a four-year history of diabetes mellitus and hypertension presented with a 15-day history of progressive orofacial symptoms, including a bulge over the hard palate, mobility of the right maxillary teeth, numbness of the upper palate, persistent bitter taste, and foul-smelling nasal discharge predominantly from the right nostril. The patient had no significant family history.

The patient initially consulted an otorhinolaryngologist. Microbiological examination of tissue obtained from the right nasal cavity by potassium hydroxide (KOH) preparation demonstrated septate branching fungal hyphae with numerous spores together with occasional broad aseptate hyphae. Subsequent fungal culture demonstrated growth of Rhizopus species and *Aspergillus fumigatus* according to the clinical microbiology laboratory report. The fungal culture was performed on a tissue specimen obtained from the right nasal cavity. The specimen was processed at Surat Pathoscan Lab. The fungal culture was performed on Sabouraud dextrose agar (SDA) and incubated under aerobic conditions at 25 °C and 37 °C. The culture report documented isolation of *Rhizopus* spp. and *Aspergillus fumigatus*. According to the microbiologist, identification of *Aspergillus fumigatus* was based on characteristic pigmentation and colony morphology, and no molecular test was performed. Identification of *Rhizopus* spp. was based on its characteristic growth pattern, colony appearance, and microscopic morphology, including the presence of broad, ribbon-like fungal hyphae. Thus, the organisms were identified based on conventional phenotypic and morphological characteristics.

Based on the clinical presentation and initial microbiological findings, invasive fungal rhinosinusitis was considered the leading diagnosis, with mucormycosis as the principal differential diagnosis and the possibility of concomitant aspergillosis also considered. Non-contrast computed tomography (CT) of the paranasal sinuses and orbits performed on 17 January 2025 demonstrated osteomyelitis involving the hard palate and bilateral maxillary alveolar processes, together with right ethmoidal and maxillary sinusitis and associated rhinitis (Figure 1A). These radiological findings were highly suggestive of an invasive rhino-maxillary fungal infection. Based on the clinical, radiological, and microbiological findings, the overall evidence supported a diagnosis of invasive rhino-maxillary mucormycosis, with concomitant *Aspergillus fumigatus* identified on culture, suggesting possible mixed fungal infection.

The treating team recommended contemporary standard-of-care management comprising functional endoscopic sinus surgery (FESS) and systemic antifungal therapy. Following discussion of the available treatment options, the patient declined the proposed interventions because of concerns regarding surgical invasiveness and the anticipated financial burden and subsequently sought consultation for Ayurveda-based management. The patient underwent stand-alone Ayurveda treatment while continuing biomedical follow-up and treatment for diabetes mellitus.

The patient demonstrated progressive clinical improvement throughout follow-up, with complete resolution of the palatal swelling after approximately 150 days of treatment (Table 1). A follow-up 96-slice CT scan (0.75 mm contiguous sections) of the paranasal sinuses and orbits performed after approximately 11 months of Ayurveda treatment was interpreted by the reporting radiologist as demonstrating complete resolution of sinusitis, stabilisation of the previously identified osseous changes, and radiological features suggestive of new bone formation and healing (Figure 1B).

### 3.2. Case 2

A 47-year-old male from western India presented with pain and heaviness over the left side of the head, a necrotic lesion involving the left hard palate (Figure 2A), restricted ocular movements, and blurred vision. The patient’s medical history was significant for uncontrolled diabetes mellitus, hypertension, chronic kidney disease, previous chikungunya infection, and a solitary functioning kidney. Baseline laboratory investigations demonstrated elevated serum creatinine and reduced estimated glomerular filtration rate.

Plain and contrast-enhanced magnetic resonance imaging (MRI) of the brain and paranasal sinuses, performed with intravenous (IV) gadolinium contrast on 1 March 2024, demonstrated invasive fungal involvement of the left ethmoidal sinus with extension to the hard palate, skull-base osteomyelitis, surrounding inflammatory soft-tissue changes, the black turbinate sign, and perineural spread along the ophthalmic and maxillary divisions of the left trigeminal nerve (Figure 3A).

Potassium hydroxide examination performed on 2 March 2024 demonstrated zygomycete fungal hyphae, while subsequent fungal cultures of palatal and sinonasal specimens yielded Mucor species and *Candida krusei*, as reported by the clinical microbiology laboratory. The specimen was examined at Dr. Bhatt Pathology Laboratory. KOH examination demonstrated broad, aseptate fungal hyphae resembling Zygomycetes/Mucor. Fungal culture was performed on Sabouraud dextrose agar (SDA) under aerobic conditions at 25 °C and 35–37 °C. The culture showed scanty growth of *Mucor* spp. and *Candida krusei*. According to the laboratory report, identification and sensitivity testing were performed using automated Vitek 2 Compact and conventional methods. This mixed presentation may indicate concomitant fungal colonisation or mixed infection.

Based on the compatible clinical presentation, characteristic radiological findings, direct microscopy demonstrating zygomycete hyphae, and culture yielding Mucor species, the overall findings supported a diagnosis of rhino-orbital mucormycosis with skull-base involvement. The biomedical treating team suggested debridement together with systemic antifungal therapy. However, after discussion of the proposed management and considering the anticipated surgical risks in the presence of multiple comorbidities, the patient elected to undergo stand-alone Ayurveda treatment. At the initial Ayurveda consultation, the patient’s serum creatinine was 1.48 mg/dL, with an estimated glomerular filtration rate of approximately 54 mL/min/1.73 m^2^. The patient was advised to continue biomedical management and regular follow-up for diabetes mellitus, hypertension, and chronic kidney disease.

The patient demonstrated progressive clinical improvement within two weeks of treatment initiation (Table 1). Complete symptomatic recovery together with complete clinical healing of the palatal lesion was achieved within approximately five months (Figure 2B).

Follow-up non-contrast MRI (multiplanar, multi-echo, 1.5-T) of the brain and paranasal sinuses, performed after approximately four months of Ayurveda treatment (8 July 2024), was interpreted by the reporting radiologist as demonstrating marked reduction in inflammatory changes involving the left pterygopalatine fossa and sphenopalatine foramen, near-complete resolution of abnormalities within the retro-maxillary fat space, and residual findings considered consistent with post-inflammatory sequelae rather than active invasive infection (Figure 3B). At the nine-month follow-up, the patient remained clinically stable without evidence of disease recurrence or treatment-related adverse events. The serum creatinine concentration had increased to 3.3 mg/dL, consistent with progression of the underlying chronic kidney disease. 

### 3.3. Case 3

A 75-year-old male from western India with a 15-year history of diabetes mellitus presented with a 20-day history of nasal regurgitation of fluids during swallowing, persistent bitter taste, foul-smelling nasal discharge, and heaviness over the right side of the face.

Initial potassium hydroxide examination performed on 17 May 2022 did not demonstrate fungal elements. However, histopathological examination of maxillary sinus tissue performed on 25 May 2022 reported mucormycosis by demonstrating fungal invasion into bone.

Non-contrast multidetector computed tomography (CT) performed on 14 May 2022 demonstrated extensive mucosal thickening involving the right maxillary and frontal sinuses together with bilateral ethmoidal and sphenoidal sinuses. Additional findings included inflammatory infiltration of the right retro-antral fat space and infratemporal region with early erosive changes involving the nasal septum, suggestive of osteomyelitis. Further radiological evaluation demonstrated extensive destruction involving the floor and walls of the right maxillary sinus, resulting in oro-antral fistula formation and displaced osseous fragments within the maxillary sinus cavity, indicative of advanced local tissue destruction (Figure S1). The radiological findings, together with the histopathological examination, supported a diagnosis of invasive mucormycosis, with histopathology demonstrating fungal invasion into bone.

The patient initially underwent functional endoscopic sinus surgery (FESS) as recommended by the treating otorhinolaryngologist. Immediately following surgery, the patient sought Ayurveda treatment and declined systemic antifungal therapy. The patient subsequently underwent stand-alone Ayurveda management while continuing biomedical care for diabetes mellitus.

The patient demonstrated substantial clinical improvement during approximately 90 days of treatment (Table 1). Long-term follow-up extending to three years demonstrated sustained symptom resolution without clinical evidence of recurrence. Follow-up non-contrast multidetector computed tomography imaging performed three years after treatment (on 27 August 2025) demonstrated persistent residual mucosal thickening involving multiple paranasal sinuses. The radiology report described post-operative changes and sequelae of previous infective aetiology, with no evidence of active infective aetiology on the plain scan (Figure S1).

### 3.4. Case 4

A 75-year-old female with a 20-year history of diabetes mellitus and hypertension, and a history of cholecystectomy one month before presentation, presented with headache, facial pain and heaviness, ocular pain, and fever.

Microbiological examination of tissue specimens obtained from the right nostril on 13 March 2025 demonstrated filamentous fungal elements. Although direct smear microscopy did not clearly distinguish septate from aseptate hyphae, the findings confirmed the presence of fungal structures within the tissue specimen.

A 96-slice (0.5 mm high-resolution), non-contrast computed tomography scan of the paranasal sinuses performed on 8 March 2025 demonstrated bilateral chronic pansinusitis, more pronounced on the right side, with complete obstruction of the right osteomeatal complex (Figure 4A). Additional CT findings included diffuse rarefaction and thinning of the adjacent osseous structures, moderate polypoidal mucosal thickening involving the right frontal and ethmoidal sinuses, severe mucosal thickening involving both maxillary and sphenoidal sinuses, and destruction of the right middle turbinate. Collectively, these radiological findings were consistent with invasive fungal rhinosinusitis, with mucormycosis considered the leading differential diagnosis.

Histopathological examination of a biopsy specimen obtained from the right nasal cavity on 17 March 2025 demonstrated necrotic nasal mucosa containing broad aseptate fungal hyphae morphologically consistent with Mucorales, together with additional thin fungal hyphae and bacterial colonies. Fungal invasion of the soft tissue was evident, and no evidence of malignancy was identified. These histopathological findings supported a diagnosis of invasive fungal rhinosinusitis involving Mucorales, based on the presence of broad aseptate hyphae morphologically consistent with Mucorales and demonstrated tissue invasion. The additional thin fungal hyphae suggested concomitant fungal involvement and were therefore reported as possible mixed fungal rhinosinusitis. Considering the histopathological evidence of tissue invasion, extensive sino-nasal involvement, and radiological evidence of bony destruction, the treating team recommended conventional management.

Following discussion of the available treatment options, the patient elected to pursue Ayurveda-based management because of concerns regarding the perceived risks associated with standard therapy.

The patient underwent Ayurveda treatment and demonstrated significant improvement in most symptoms during three months of follow-up (Table 1). However, adherence to the prescribed therapeutic regimen and dietary recommendations remained suboptimal because of personal constraints. Follow-up 96-slice non-contrast computed tomography of the paranasal sinuses performed after approximately three months (13 June 2025) demonstrated persistent destructive osseous changes involving the maxilla, hard palate, sphenoid bone, clivus, and right ethmoidal region, together with residual inflammatory soft-tissue opacification involving the bilateral sphenoid, right ethmoidal, and right maxillary sinuses (Figure 4B). These findings were interpreted as post-treatment sequelae of invasive fungal rhinosinusitis without convincing radiological evidence of disease regression. The patient survived for approximately one year following diagnosis.

Because outcome data were derived from routine clinical documentation, and follow-up intervals, symptom assessment, and radiological evaluation were not standardised across cases, the observed clinical and radiological changes should be interpreted descriptively. Radiological findings were based on routine reports and were not independently or blindly reviewed.

Treatment and Diet

From an Ayurvedic perspective, the clinical presentation of the cases was interpreted in relation to the concepts of Raktaja Krimi (pathological organisms affecting blood and vascular tissues) and Asthi-Majjagata Krimi (pathological involvement of bone and marrow tissues), as described in the WHO International Standard Terminologies on Ayurveda (ITA 5.13.7; ITA 2.2.1; ITA 5.13). The treatment strategy was formulated through an integrative understanding of the contemporary clinical manifestations of mucormycosis and their Ayurvedic interpretation. A central therapeutic consideration was the regulation of kleda (moistness or pathological fluid accumulation; ITA-D.2534), which is understood to create conditions conducive to disease progression. Accordingly, both therapeutic interventions and supportive dietary measures were directed toward reducing excessive kleda and restoring physiological balance (Table S1) [22].

All patients were advised to follow a diet and lifestyle regimen designed to minimise factors considered to promote kleda accumulation. The dietary recommendations emphasised easily digestible foods, bitter vegetables, and moderate consumption of camel milk, while discouraging excessive intake of sweet, sour, heavy, and moisture-promoting foods. Lifestyle guidance included avoiding daytime sleep, prolonged exposure to cold environments, and other factors traditionally associated with increased kleda, while encouraging measures that promote warmth and metabolic regulation. Notably, Cases 1–3 demonstrated satisfactory adherence to these recommendations, whereas adherence was suboptimal in Case 4. The patient did not consistently adhere to the prescribed treatment regimen, with occasional missed doses and insufficient consumption of the prescribed oral medications. In addition, adherence to key dietary recommendations, particularly the advised consumption of camel milk, was inconsistent. Within the Ayurvedic framework, such variations in compliance may represent an important factor influencing therapeutic outcomes and warrant further consideration in future clinical investigations. Dietary and lifestyle recommendations based on the Ayurvedic concept of kleda reduction were incorporated as supportive components of the individualised management approach and should be interpreted separately from the primary therapeutic interventions. As this retrospective case series did not employ standardised methods to assess adherence or evaluate the independent contribution of these recommendations, no causal relationship between adherence and clinical outcomes can be inferred.

This retrospective descriptive case series was based exclusively on anonymized clinical data obtained during routine patient care. No additional intervention, investigation, patient recruitment, or interaction was undertaken for research purposes. In accordance with the applicable institutional policy and the ICMR National Ethical Guidelines (2017), formal Ethics Committee approval was not required. Written informed consent for publication was obtained from all patients or their legally accepted representatives, as applicable [23].

Given the potentially life-threatening nature of the infection, patients and/or their legally accepted representatives were counselled regarding the seriousness of the condition, available conventional treatment options, including amphotericin-based antifungal therapy and surgical debridement where clinically indicated, and the potential risks associated with refusing or delaying such treatment, including disease progression, orbital or intracranial extension, irreversible morbidity, disfigurement, and death. The clinical counselling and treatment decision-making process were undertaken as part of routine patient care and was distinct from the written informed consent obtained for publication of anonymized clinical information.

Safety information was retrospectively obtained from available clinical records, laboratory investigations, treatment documentation, and follow-up records. As this was a retrospective case series, no predefined adverse-event assessment protocol or standardised laboratory monitoring schedule was used.

An adverse event (AE) was defined as any untoward medical occurrence during treatment or follow-up, irrespective of its relationship to the intervention. A serious adverse event (SAE) included events resulting in death, life-threatening conditions, hospitalisation or its prolongation, significant disability, or other medically important conditions. Clinically significant events were those requiring medical intervention or treatment modification or resulting in persistent symptoms or organ dysfunction.

Where sufficient information was available, the relationship of an event to treatment was retrospectively assessed considering temporal association, comorbidities, alternative explanations, and concomitant interventions, and categorised as unrelated/unlikely, possible, probable, or indeterminate. Renal and hepatic parameters were monitored as part of routine clinical care at variable intervals; therefore, available laboratory findings represent routine clinical observations rather than a standardised pharmacovigilance assessment.

## 4. Discussion

The present case series describes the clinical course, radiological evolution, and long-term outcomes of four cases of mucormycosis managed predominantly through Ayurveda-based interventions following diagnosis by conventional biomedical investigations (Table 2). To our knowledge, this represents one of the first published case series documenting predominantly Ayurveda-based management of mucormycosis with comprehensive clinical, radiological, and long-term follow-up.

The cohort represented a spectrum of advanced craniofacial mucormycosis, comprising three patients with rhino-maxillary disease (including cases with maxillary osteomyelitis and skull-base extension) and one patient with rhino-orbital disease complicated by skull-base involvement and perineural spread. Two patients also demonstrated concomitant mixed fungal infections involving *Aspergillus* spp. or *Candida* spp. Among the four patients, three showed clinical improvement accompanied by radiological changes interpreted by the reporting radiologists as improvement during follow-up ranging from nine months to four years (File S1). No clinical or radiological evidence of disease recurrence was documented during the respective follow-up periods in these three patients. An important observation was the clinical improvement and documented radiological changes in the patient with skull-base mucormycosis and perineural spread (Case 2). Skull-base involvement represents one of the most aggressive manifestations of mucormycosis and is frequently associated with extensive tissue invasion, neurological complications, and poor clinical outcomes [24]. Such patients generally require aggressive multimodal management consisting of prolonged systemic antifungal therapy together with extensive surgical debridement and, in selected cases, repeated surgical procedures to achieve disease control [25]. Against this clinical background, the clinical improvement and documented radiological changes over approximately nine months following predominantly Ayurveda-based management merit further investigation. Although causal inferences cannot be drawn from an uncontrolled case series, this clinical observation highlights the need for systematic evaluation of Ayurveda-based therapeutic strategies in advanced mucormycosis through appropriately designed clinical studies.

Case 4 demonstrated a comparatively different clinical trajectory. The patient experienced moderate symptomatic improvement during the initial three months of treatment; however, follow-up computed tomography did not demonstrate corresponding radiological resolution. Interpretation of this observation requires caution because treatment adherence became suboptimal after the initial treatment period, potentially influencing therapeutic response. Although convincing radiological improvement was not demonstrated, the patient’s survival for approximately one year remains a clinically relevant descriptive outcome, particularly as three-month survival is a widely reported endpoint in mucormycosis research [26].

Furthermore, persistent radiological abnormalities identified approximately three months after treatment initiation should not be interpreted as definitive evidence of treatment failure, since radiological resolution of invasive fungal rhino sinusitis may lag behind clinical recovery. A follow-up imaging assessment performed after five to six months under satisfactory treatment adherence may have provided a more reliable evaluation of disease evolution. In this case, clinical records documented substantially reduced medicine consumption followed by treatment discontinuation during the course of management. This observation is reported descriptively as one of several factors that may have influenced the overall clinical course, alongside disease severity, baseline clinical characteristics, concomitant management, and other unmeasured variables. Accordingly, no causal attribution is made between treatment adherence and the observed outcome. Collectively, these findings highlight the importance of standardised radiological follow-up protocols in future Ayurveda-based mucormycosis studies, while accounting for baseline disease severity, treatment adherence, and temporal changes in radiological response.

The diagnosis of mucormycosis in this case series was established through a comprehensive multidisciplinary assessment integrating clinical presentation, radiological findings, microbiological investigations, and histopathological examination where available. Although the EORTC/MSGERC consensus definitions consider histopathological demonstration of tissue invasion a key criterion for the classification of proven mucormycosis, these definitions were primarily developed to standardise patient enrolment in epidemiological studies, clinical trials, and diagnostic research rather than to direct routine clinical decision-making [27]. As acknowledged by the consensus update, these research definitions are not intended to serve as the sole framework for day-to-day clinical diagnosis. Accordingly, this descriptive case series reflects real-world clinical practice, in which diagnostic decisions were based on multidisciplinary integration of compatible clinical features, characteristic radiological findings, microbiological evidence from tissue specimens, and histopathological confirmation where available. This pragmatic diagnostic approach is consistent with contemporary clinical management of mucormycosis while transparently presenting the diagnostic evidence supporting each individual case. Diabetes mellitus was present in all four patients, consistent with its well-established role as the predominant predisposing factor for mucormycosis, particularly in low- and middle-income countries [4]. One of the patients (Case 2) had CKD, which is considered an emerging risk factor [28]. Additional comorbidities included hypertension, previous chikungunya infection, and a solitary functioning kidney, thereby reflecting the complex clinical profile frequently encountered in routine practice. Consequently, this case series provides preliminary documentation regarding the clinical course, radiological outcomes, and long-term follow-up associated with predominantly Ayurveda-based management in patients with mucormycosis presenting with multiple high-risk clinical characteristics (Table 2).

The findings should also be interpreted within the broader healthcare context of low- and middle-income countries, where delayed diagnosis, challenges in achieving optimal glycaemic control, limited access to specialised centres, and financial constraints frequently contribute to advanced disease at presentation and poorer clinical outcomes [29,30]. Current evidence consistently identifies early initiation of systemic antifungal therapy together with prompt surgical debridement as the cornerstone of mucormycosis management, while delayed intervention has repeatedly been associated with increased mortality [31]. Boqaaiya et al. and other investigators have similarly emphasized that timely antifungal therapy and appropriate surgical intervention are critical determinants of survival [32]. Despite these established recommendations, mortality and substantial morbidity continue to be reported even in tertiary-care settings employing aggressive multimodal treatment strategies.

Against this background, the observations from the present series warrant careful consideration. All patients had already undergone comprehensive biomedical evaluation and diagnostic confirmation before initiating Ayurveda-based management, and most sought Ayurveda relatively late in the disease course after conventional treatment options had been discussed. Nevertheless, three patients demonstrated clinical improvement accompanied by radiological changes documented on serial follow-up imaging, as interpreted in the respective radiology reports.

Although these observations should not be interpreted as evidence of therapeutic equivalence or superiority to contemporary standard-of-care management, they generate clinically relevant hypotheses for future research evaluating predominantly Ayurveda-based management in carefully selected clinical contexts.

These preliminary findings support the need for prospective observational studies and appropriately designed comparative clinical investigations to evaluate efficacy, safety, patient-centred outcomes, and to determine whether any role exists for integration alongside current standards of care.

An additional observation arising from the present series relates to the predominantly non-invasive therapeutic approach employed. Surgical management of mucormycosis, although considered an essential component of contemporary standard-of-care treatment, is frequently associated with substantial functional and cosmetic sequelae, including extensive tissue loss, craniofacial deformity, visual impairment, and neurological deficits, depending on disease extent [33]. Recent evidence has suggested that organ-preserving and minimally invasive endoscopic approaches may achieve satisfactory disease control in selected patients with rhino-orbito-cerebral mucormycosis, highlighting that successful management may depend not only on radical surgical excision but also on effective reduction in fungal burden [34]. Within this context, the present observations generate hypotheses for future research exploring whether predominantly Ayurveda-based interventions could be evaluated as adjuncts to contemporary management, including organ-preserving surgical approaches where clinically appropriate. Such hypotheses require rigorous clinical investigation and should not be interpreted as supporting modification of current standard-of-care management.

Safety remains a critical consideration in the evaluation of any therapeutic strategy for mucormycosis. Systemic antifungal agents, particularly amphotericin B, are well recognised for their association with nephrotoxicity, electrolyte disturbances, and infusion-related adverse reactions [8]. In the present series, no treatment-related adverse events were documented in the available records (Table 3). Available biochemical monitoring in Cases 1 and 3 did not demonstrate substantial deterioration in hepatic or renal parameters. In contrast, Case 2 had substantial baseline renal risk, including a solitary functioning kidney, chronic kidney disease/chronic renal failure documented at baseline by the treating consultant, treatment-resistant hypertension, and impaired renal function before initiation of Ayurveda-based management. Serum creatinine increased from 1.48 mg/dL at baseline to 3.3 mg/dL during follow-up. Although the patient had documented pre-existing chronic renal impairment and multiple risk factors for progressive renal dysfunction, the observed increase in serum creatinine represents an important safety concern. Within the limitations of this uncontrolled case series, the relative contribution of progression of underlying renal disease and a potential treatment-related effect cannot be determined, and a treatment-related contribution cannot be completely excluded. This uncertainty is particularly relevant given the use of herbo-mineral formulations. These observations underscore the importance of standardised longitudinal monitoring of renal and hepatic function and careful safety assessment in future clinical investigations evaluating Ayurveda-based management, particularly among patients with pre-existing renal impairment or other high-risk comorbidities.

The use of herbo-mineral formulations in Case 2 should be interpreted within the context of advanced skull-base mucormycosis, a rapidly progressive and potentially fatal infection associated with a limited therapeutic window. Within the Ayurveda-based treatment strategy adopted for this patient, herbo-mineral preparations constituted an essential component of the treatment approach. Their use was undertaken in the context of the severity of the underlying disease and the patient’s pre-existing renal risk factors. Clinical management incorporated several risk-mitigation measures, including continuation of conventional management for hypertension, routine biomedical follow-up, available biochemical monitoring, and clinical observation documented during treatment. The formulations were manufactured by licensed GMP-certified pharmaceutical facilities, and informed consent addressed the proposed treatment, potential risks, therapeutic uncertainties, and criteria for referral in the event of clinical deterioration. However, these measures were not implemented within a predefined prospective safety-monitoring protocol, and adverse-event assessment remained retrospective, non-standardised, and dependent on available clinical documentation. Therefore, they should not be interpreted as establishing the safety of the treatment approach.

Intranasal fumigation was administered at a distance of approximately 30–45 cm from the patient’s nostrils, allowing cooled and diluted fumes to reach the nasal passages while avoiding direct exposure to heat or concentrated smoke. The exposure was intermittent and delivered as a gentle, controlled flow rather than continuous or excessive inhalation. Clinical observations recorded during administration included monitoring for potential local and respiratory symptoms such as nasal or throat irritation, burning sensation of the eyes or throat, breathlessness, worsening nasal symptoms, and other respiratory discomfort. However, these observations were based on routine clinical documentation rather than a predefined or standardised adverse-event assessment protocol.

Transient symptoms such as mild throat or ocular irritation or mild breathlessness were documented following exposure in some instances and generally resolved spontaneously within approximately 5–10 min without requiring medical intervention. No clinically significant mucosal burns, persistent airway symptoms, secondary respiratory infections, or sustained worsening of local inflammatory symptoms were documented in the available clinical records during the treatment period or subsequent reported follow-up. However, because adverse-event monitoring was retrospective, non-standardised, and dependent on routine clinical documentation, the absence of documented clinically significant adverse events should not be interpreted as evidence establishing the safety of intranasal fumigation. Patients with severe underlying respiratory illness were not administered this intervention.

The available human evidence concerning the safety of Ayurvedic nasal fumigation remains limited. A recent pilot clinical study evaluated polyherbal Dhoopana in children with rhinitis, and a subsequently published clinical trial protocol describes structured administration and adverse-event monitoring; however, these studies concern substantially different clinical populations and indications and cannot be extrapolated to establish the safety of fumigation in patients with invasive fungal rhinosinusitis [35,36].

Accordingly, the present observations should not be interpreted as establishing the safety of intranasal fumigation or the broader Ayurveda-based treatment approach. The safety observations reported here are limited to adverse effects documented in the available clinical records of this small retrospective case series. Baseline liver function measurements were unavailable, adverse-event monitoring was retrospective and non-standardised, and the completeness of clinical documentation could not be independently verified. Larger prospective studies incorporating predefined adverse-event criteria, standardised exposure parameters, baseline and longitudinal renal and hepatic assessments, and systematic assessment of nasal, ocular, respiratory, and systemic effects are required to characterise the safety profile of these interventions more reliably.

Although herbo-mineral formulations may pose safety concerns when manufacturing quality is inadequate or when they are administered inappropriately, the formulations employed in the present series were produced by licensed GMP-certified pharmaceutical facilities and prescribed according to recognised Ayurvedic clinical practice (Table S1). Published toxicological and clinical investigations have suggested that some properly manufactured herbo-mineral formulations prepared using standardised purification and processing procedures described in classical Ayurveda may have acceptable safety profiles under defined conditions and therapeutic dosages [37]. Similarly, the only previously published report describing stand-alone Ayurveda-based management of mucormycosis documented normal renal and hepatic parameters during the reported treatment period despite the use of comparable formulations [21]. However, these observations are insufficient to establish safety, particularly in the absence of adequately designed prospective studies and standardised pharmacovigilance. They should therefore be interpreted only as preliminary observations supporting the need for rigorous prospective pharmacovigilance and systematic safety evaluation in future clinical research.

The financial burden of mucormycosis management represents an important consideration, particularly within resource-constrained healthcare systems. An earlier observational study from India observed that a considerable proportion of patients left standard biomedical treatment against medical advice, with economic hardship frequently cited as the reason for treatment discontinuation [38]. All four patients reported that the anticipated cost associated with conventional treatment influenced their therapeutic decision-making and contributed to their preference for Ayurveda-based care. Although the present case series did not include a formal economic evaluation, the consistent observation of cost as an important consideration in treatment choice highlights the potential relevance of economic factors in healthcare decision-making among patients with this condition. This finding warrants further investigation through appropriately designed comparative economic evaluations, including cost-effectiveness, cost-minimization, or cost-utility analyses, to systematically compare Ayurveda-based and conventional biomedical care pathways.

The biological plausibility of Ayurveda-based therapeutic approaches has been explored in experimental research evaluating the pharmacological properties of individual medicinal plants and formulations used in Ayurvedic practice. Laboratory investigations, including computational, in vitro, and animal studies, have reported antifungal, immunomodulatory, and anti-inflammatory activities for several Ayurvedic botanicals against selected fungal pathogens or inflammatory targets, although these findings largely derive from preclinical models rather than studies of invasive mucormycosis specifically [39,40].

Experimental evaluation of an Ayurvedic intranasal formulation (Anu Taila) demonstrated inhibitory activity against Mucor spores and modulation of TNF-α-associated inflammatory pathways in laboratory models [41]. Several medicinal plants incorporated into the individualised treatment protocols in the present case series, including *Embelia ribes* (Vidanga), *Butea monosperma* (Palash), *Semecarpus anacardium* (Bhallataka), *Albizia lebbeck* (Shirisha), and *Azadirachta indica* (Neem), were also components of the stand-alone Ayurveda-based treatment previously reported for mucormycosis [21]. Experimental studies have demonstrated antifungal activity for extracts of E. ribes and A. lebbeck, while embelin, the principal constituent of E. ribes, has shown inhibition of TNF-α-converting enzyme and modulation of NF-κB signalling pathways under experimental conditions [42,43,44,45]. These findings identify biological activities of individual constituents but do not establish the therapeutic effects of the individualised formulations used in this case series.

Collectively, these experimental observations provide hypotheses that may justify further mechanistic investigation but should not be interpreted as evidence of clinical efficacy in invasive mucormycosis. Establishing biological activity requires dedicated in vitro studies against Mucorales, appropriate animal models, and translational pharmacological investigations.

Clinical evidence supporting Ayurveda in mucormycosis remains limited. Madikonda et al. reported favourable outcomes in a non-randomised study involving 92 patients, in which Ayurveda was used as an adjunct to conventional management, with outcomes including survival, recurrence, requirement for advanced surgical procedures, and overall clinical improvement [46]. In contrast, the present case series describes predominantly Ayurveda-based management and includes serial radiological observations during extended follow-up, which were not major outcome measures in the previous study.

Rajnikant et al. subsequently reported a single case of predominantly Ayurveda-based management of COVID-19-associated rhino-orbital mucormycosis with radiological resolution [21]. The present series advances the existing descriptive reporting by documenting multiple cases with heterogeneous and clinically challenging presentations, while providing additional clinical, radiological, and longitudinal observations. These observations extend and advance hypothesis generation rather than establishing treatment effectiveness.

The findings should also be interpreted within the broader methodological landscape of mucormycosis research. The rarity of the disease, rapid clinical progression, marked heterogeneity in patient characteristics, and ethical challenges associated with withholding established treatment substantially limit the feasibility of adequately powered randomised controlled trials. Consequently, well-documented observational studies and real-world clinical evidence remain important sources of information for hypothesis generation, refinement of outcome measures, and development of future clinical research. Within this context, the present series contributes preliminary evidence regarding feasibility of adapting clinical outcomes, radiological assessment, long-term follow-up, and safety monitoring associated with predominantly Ayurveda-based management.

Nevertheless, these findings should be interpreted cautiously and should not be construed as evidence supporting generalised treatment recommendations or therapeutic equivalence with contemporary standard-of-care management. Rather, this case series provides hypothesis-advancing evidence that may inform future prospective observational studies and carefully designed comparative clinical investigations. Importantly, the study demonstrates the feasibility of applying conventional biomedical outcome measures, including survival, case-specific diagnostic evidence, serial radiological assessment, and longitudinal safety monitoring, to evaluate Ayurveda-based interventions. Collectively, these observations contribute methodological insights that may facilitate the development of scientifically rigorous, ethically appropriate, and contextually relevant research protocols for investigating the role of Ayurveda within the multidisciplinary management of mucormycosis.

## 5. Patient Perspectives

Case 1: The patient reported concerns regarding potential facial disfigurement associated with surgery and the anticipated financial burden of conventional treatment. During follow-up, the patient expressed satisfaction with the recovery and the non-surgical management received.

Case 2: The patient reported similar concerns regarding surgery and treatment-related costs and stated that a recommendation from a previously treated patient influenced the decision to seek Ayurveda care. During follow-up, the patient expressed satisfaction with the clinical outcome.

Case 3: The patient described anxiety regarding surgery and concerns about financial constraints before treatment. During follow-up, the patient reported satisfaction with the clinical improvement achieved.

A patient perspective statement was not available for Case 4.

## 6. Limitations

The findings of this case series should be interpreted in light of several limitations. Follow-up assessments were not conducted at standardised time points, resulting in variability in the timing of clinical, radiological, and laboratory evaluations across cases. This limitation restricted systematic comparison of treatment trajectories and highlights the need for standardised follow-up schedules, particularly for radiological assessment within clinically relevant time windows. Batch-level quality-control data for the formulations used in these cases, including analytical data on heavy-metal content, were not available. Therefore, formulation-specific quality and compositional characteristics could not be independently verified, representing an additional limitation when interpreting the safety observations. Similarly, a predefined safety monitoring framework, including uniform kidney and liver function testing intervals, was not implemented, which is especially relevant for patients with baseline renal impairment or other high-risk comorbidities. Comprehensive ophthalmological outcome assessment was also limited, as formal visual field evaluation was not available for Case 2. The symptom assessment presented in this case series is based on patient-reported outcomes rather than standardised assessment. Microbiological assays and methods used to identify or characterise the fungi were not conclusive, and molecular methods would be needed to confirm the presumed pathogenic organisms.

## 7. Conclusions

This case series describes four cases of mucormycosis managed predominantly through Ayurveda-based interventions, with comprehensive clinical, radiological, and long-term follow-up. Three patients showed clinical improvement accompanied by radiological changes interpreted by the reporting radiologists as improvement during follow-up periods ranging from 9 months to 4 years, without documented disease recurrence. One patient demonstrated persistent radiological abnormalities at the three-month assessment in the context of suboptimal treatment adherence and subsequently discontinued treatment, although survival was maintained for approximately one year following diagnosis. No treatment-related adverse events were documented in the available follow-up records. One patient with pre-existing chronic kidney disease exhibited progressive deterioration in renal function during follow-up; although the clinical course appeared more consistent with progression of the underlying renal disease, a treatment-related contribution could not be definitively excluded. These observations should be interpreted as descriptive findings from an uncontrolled case series with non-standardised outcome assessment.

Although the findings should not be interpreted as evidence of treatment effectiveness or as support for generalised clinical recommendations, they provide descriptive clinical observations that extend and advance existing hypothesis generation regarding predominantly Ayurveda-based management of mucormycosis. To our knowledge, this is one of the first case series to comprehensively document predominantly Ayurveda-based management with case-specific diagnostic evidence, serial radiological assessment, and prolonged follow-up across patients with diverse clinical presentations, including advanced skull-base disease.

Beyond its clinical observations, this paper provides methodological insights for future investigations by demonstrating the feasibility of incorporating conventional biomedical outcome measures, including survival, radiological findings, case-specific diagnostic evidence, and longitudinal safety monitoring, into the evaluation of Ayurveda-based interventions. These observations support the need for well-designed clinical studies to evaluate clinical outcomes, safety, and patient-centred outcomes and to determine whether any role exists for Ayurveda-based interventions within multidisciplinary management strategies for mucormycosis while contemporary antifungal therapy and surgical management remain the current standard of care.

## Figures and Tables

**Figure 1:**
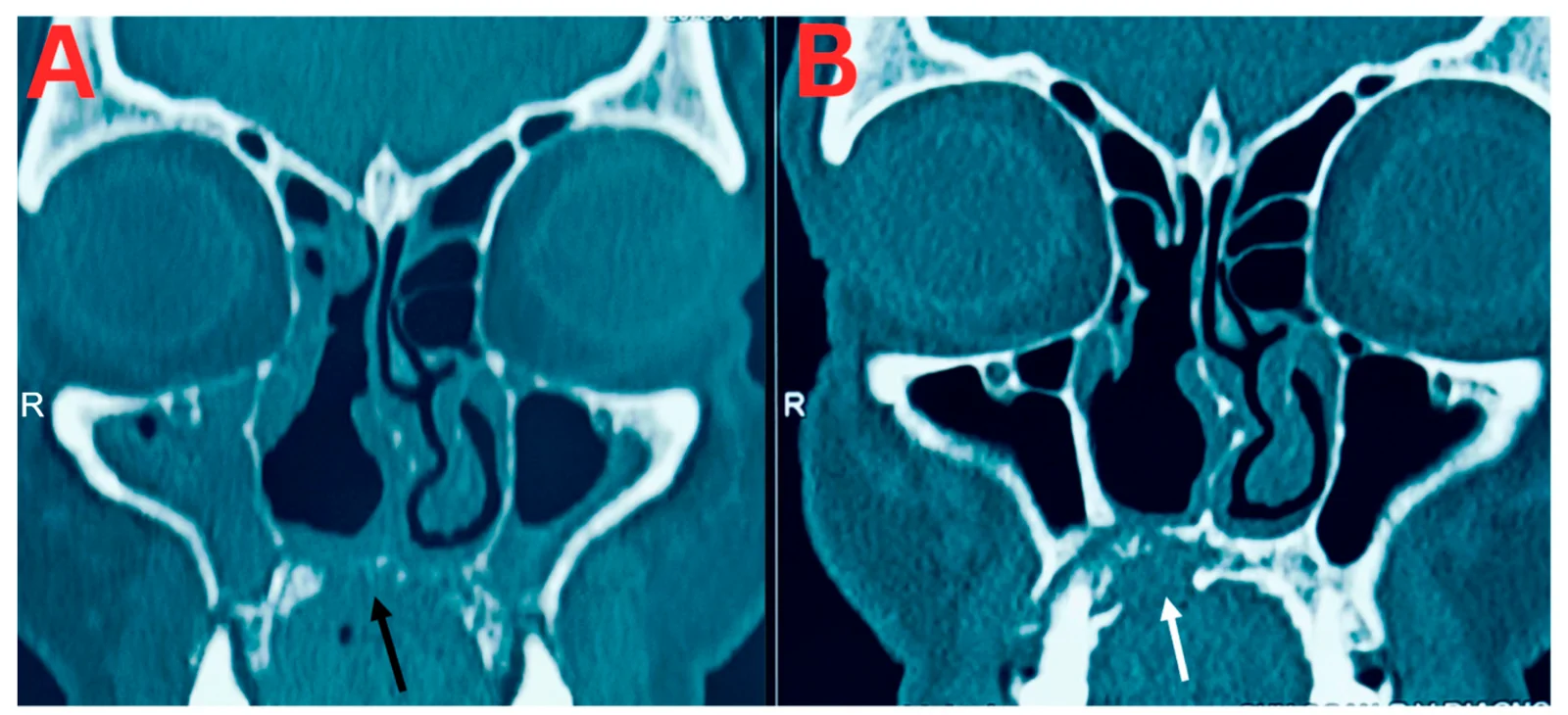
**Imaging findings before (A) and after (B) Ayurveda treatment**. (**A**) Baseline image showing significant osseous erosion and destruction involving the bilateral posterior hard palate and adjacent posterior maxillary alveolar processes, with an associated paraosseous soft-tissue component (black arrow). (**B**) Follow-up image demonstrating interval increased sclerosis, regression of the paraosseous soft-tissue component in the bilateral posterior hard palate region, and increased features of osteitis (white arrow).

**Figure 2:**
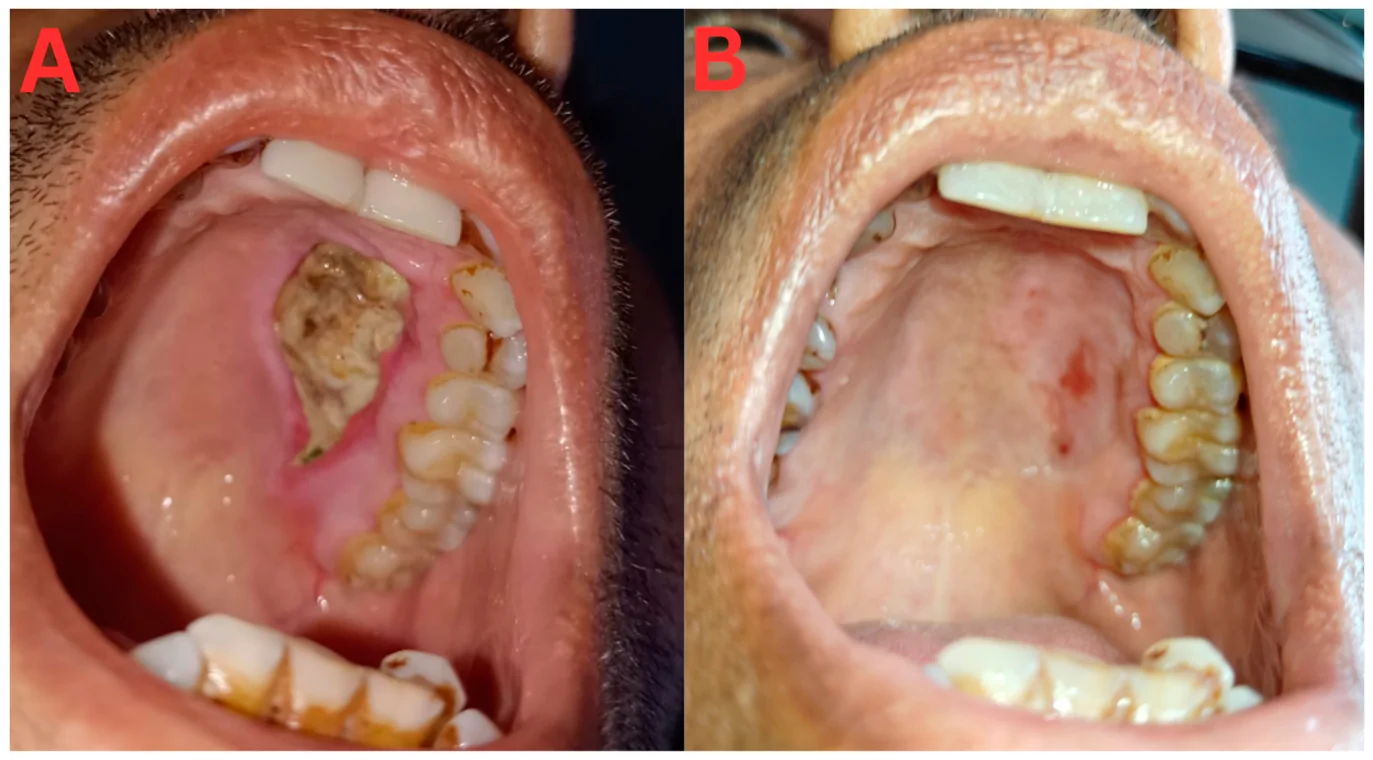
(**A**) Necrotic lesion in the hard palate. (**B**) Complete healing of the hard palate lesion.

**Figure 3:**
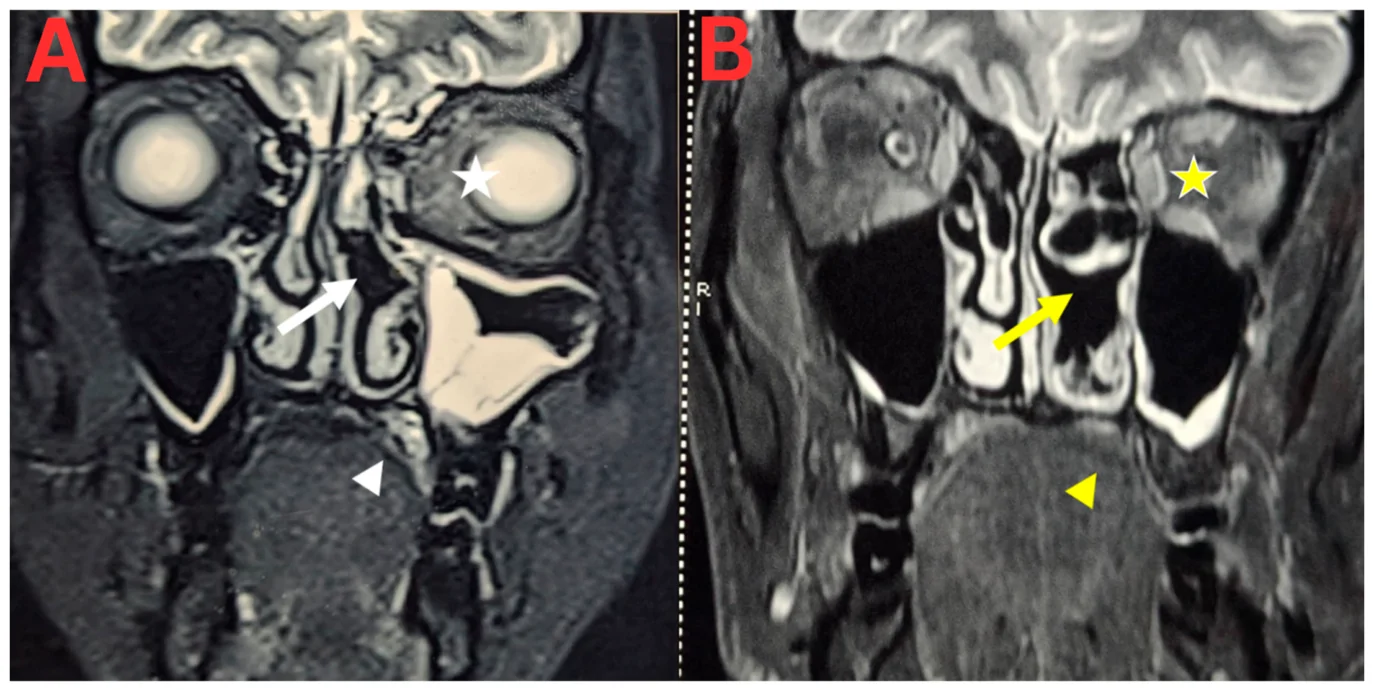
(**A**,**B**) MRI image before (**A**) and after (**B**) Ayurveda treatment. (**A**) Baseline MRI showing the left middle turbinate “*black turbinate*” *sign* (white arrow), diffuse oedematous changes and soft-tissue thickening involving the medial intraconal compartment of the left orbit (white star), and diffuse marrow oedema with adjacent soft-tissue involvement in the left posterior hard palate region (white arrowhead). (**B**) Follow-up MRI showing autologous extirpation of the previously involved left middle turbinate (yellow arrow), resolution of the previously noted soft-tissue involvement in the medial intraconal compartment of the left orbit (yellow star), and near-complete resolution of the previously noted marrow oedema and adjacent soft-tissue involvement in the left posterior hard palate region (yellow arrowhead).

**Figure 4:**
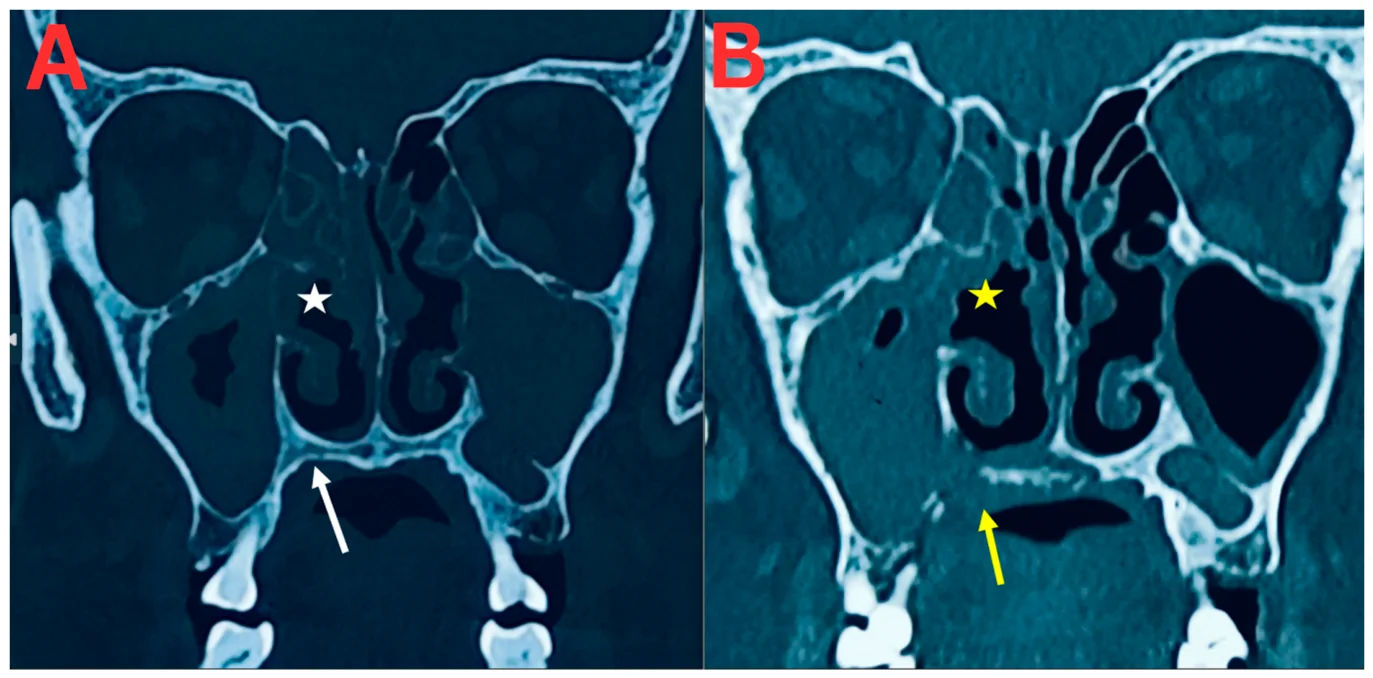
(**A**,**B**) CT image before (**A**) and after (**B**) Ayurveda treatment. (**A**) Baseline CT image showing subtle rarefaction of the right middle turbinate (white star) with polypoidal mucosal thickening in the right middle meatus. No evidence of osseous destruction involving the right hard palate is seen (white arrow). (**B**) Follow-up CT image showing an interval increase in osseous destruction of the right middle meatus with associated mucosal irregularities (yellow star), along with a significant interval increase in osseous destruction of the right posterior hard palate and the adjacent aspect of the right maxillary bone (yellow arrow).

**Table 1:** Follow-up table.

Follow-Up	Case 1	Case 2	Case 3	Case 4
1st consultation	• Mobile teeth in upper right side • Numbness in upper palate and teeth • Feeling of bitter taste in mouth • Foul smell from nostrils (right > left) • Bulging of hard palate—15 days (17/01/2025)	Pain in left side of nose, lips and eye Severe headache Numbness in left side of nose, palate, lips, eye region No perception of smell No movement of left eyeball Blurred vision in left eye O/E- Large lesion at upper palate Since 8 days (10/03/2024)	• Nasal regurgitation of fluids during oral intake • Feeling of bitter taste in mouth • Foul smell from nostrils • Heaviness of right side of face For 20 days (02/06/2022)	Headache, facial pain and heaviness, ocular pain and heaviness, and fever. For 10 days (13/03/2025)
2nd visit	• Mobile teeth in upper right side: still present • Numbness in upper palate and teeth: 40% improvement • Feeling of bitter taste in mouth: 50% improvement • Foul smell from nostrils (right > left): 50% improvement Bulging of hard palate: 15 days: slightly reduced (16/02/2025)	Improvement in pain: 20% Improvement in numbness: 20% (18/03/2024)	• Nasal regurgitation of fluids during oral intake: as it is • Feeling of bitter taste in mouth: 30% improvement • Foul smell from nostrils: 50% improvement • Heaviness of right side of face: 100% improvement (29/06/2022)	Headache: 30% improvement Facial pain and heaviness: 30% improvement Ocular pain: as it is Fever: Not there (25/03/2025)
3rd Visit	• Mobile teeth in upper right side: 30% improvement • Numbness in upper palate and teeth: 60% improvement • Feeling of bitter taste in mouth: 70% improvement • Foul smell from nostrils (right > left): 70% improvement Bulging of hard palate: 15 days: 40% improvement (23/03/2025)	Improvement in pain: 40% Improvement in numbness: 40% Improvement in smell perception: 40% Movement of left eyeball: 30% Improvement in vision: 10% (01/04/2024)	• Nasal regurgitation of fluids during oral intake: as it is • Feeling of bitter taste in mouth: 70% improvement • Foul smell from nostrils: 80% improvement (20/07/2022)	Headache: 70% improvement Facial pain and heaviness: 50% improvement Ocular pain:20% improvement (21/04/2025)
4TH VISIT	• Mobile teeth in upper right side: 60% improvement • Numbness in upper palate and teeth: 100% improvement • Feeling of bitter taste in mouth: 100% improvement • Foul smell from nostrils (right > left): 100% improvement Bulging of hard palate: 15 days: 70% improvement (17/04/2025)	Improvement in pain: 70% Improvement in numbness: 70% Improvement in smell perception: 80% Movement of left eyeball: 60% Improvement in vision: 40% O/E Improvement in lesion at upper palate: 40% (04/05/2024)	• Nasal regurgitation of fluids during oral intake: as it is • Feeling of bitter taste in mouth: 100% improvement • Foul smell from nostrils 100% improvement (19/08/2022)	Headache: 70% improvement Facial pain and heaviness: 50% improvement Ocular pain: 20% improvement (06/05/2025)
5th Visit	• Mobile teeth in upper right side: 90% improvement Bulging of hard palate: 15 days: 90% improvement (26/05/2025)	Improvement in pain: 90% Improvement in numbness: 90% Improvement in smell perception: 90% Movement of left eyeball: 75% Improvement in vision: 60% (04/06/2024)	• Nasal regurgitation of fluids during oral intake: as it is (15/09/2022)	Headache: 80% improvement Facial pain and heaviness: 70% improvement Ocular pain: 50% improvement (13/06/2025)
6th Visit	• Mobile teeth in upper right side:100% improvement • Bulging of hard palate: 100% improvement (16/06/2025)	Improvement in pain: 100% Improvement in numbness: 100% Improvement in smell perception: 100% Movement of left eyeball: 80% Improvement in vision: 70% o/e Lesion almost healed (08/07/2024)	Nasal regurgitation of fluids during oral intake reduced up to 50–60% (28/08/2025)	
7th Visit		Movement of left eyeball: 100% Improvement in vision: 80% (08/08/2024)		
8th Visit		Improvement in vision: 90–95% (07/09/2024)		
9th Visit		Improvement in vision: 100% (05/10/2024)		

This table presents follow-up information on clinical status based on non-standardised, patient-reported outcomes.

**Table 2:** Clinical summary.

Particulars	Case 1	Case 2	Case 3	Case 4
Age/Sex	65/M	47/M	75/M	75/F
Comorbidities	Diabetes mellitus, Hypertension	Diabetes mellitus, Hypertension, Chronic kidney disease, Solitary functioning kidney	Diabetes mellitus	Diabetes mellitus, Hypertension
Site and extent of disease	Rhino-maxillary mucormycosis involving hard palate, bilateral posterior maxillary alveolar process, right ethmoid and maxillary sinus with osteomyelitis	Rhino-orbital mucormycosis with skull-base osteomyelitis, ethmoid sinus, hard palate, pterygopalatine fossa, orbit and perineural spread	Rhino-maxillary mucormycosis with extensive maxillary sinus destruction, oro-antral fistula and osteomyelitis	Invasive fungal rhinosinusitis involving bilateral paranasal sinuses with destruction of the right middle turbinate and subsequent involvement of the maxilla, hard palate, sphenoid bone, clivus and right ethmoidal region.
Diagnostic evidence	Positive KOH microscopy, fungal culture, contrast-enhanced CT findings consistent with invasive fungal infection	Positive KOH microscopy, fungal culture, MRI findings characteristic of ROCM	Negative KOH microscopy, histopathology demonstrating fungal invasion into bone, and CT findings consistent with invasive fungal disease.	Positive tissue microscopy showing fungal structures, histopathology demonstrating broad aseptate hyphae with tissue invasion, and CT findings consistent with invasive fungal rhinosinusitis.
Organism identified	*Rhizopus* spp. and *Aspergillus fumigatus*	*Mucor* spp. and *Candida krusei*	Histopathology consistent with Mucorales (species not identified)	Broad aseptate hyphae consistent with Mucorales with additional thin fungal hyphae (mixed fungal infection suspected)
Standard treatment recommended	Functional endoscopic sinus surgery (FESS) with systemic liposomal amphotericin B	Surgical debridement with systemic liposomal amphotericin B	FESS followed by systemic liposomal amphotericin B	Surgical debridement with systemic liposomal amphotericin B
Treatment received before Ayurveda	None	None	FESS (declined antifungal therapy)	None
Ayurveda intervention	Predominantly Ayurveda-based treatment (oral formulations, intranasal therapy, fumigation, dietary and lifestyle advice)	Predominantly Ayurveda-based treatment (oral formulations, intranasal therapy, fumigation, dietary and lifestyle advice)	Predominantly Ayurveda-based treatment after FESS (oral formulations, intranasal therapy, fumigation, dietary and lifestyle advice)	Predominantly Ayurveda-based treatment (oral formulations, intranasal therapy, fumigation, dietary and lifestyle advice)
Follow-up duration	17 months	9 months	4 years	1 year
Radiological outcome	Resolution of sinusitis with sclerosis and healing suggestive of new bone formation	Marked reduction in inflammatory changes with residual post-inflammatory sequelae; no radiological evidence of active invasive disease	Persistent residual mucosal thickening without radiological evidence of active invasive fungal disease or progression	Persistent destructive osseous changes without convincing radiological regression
Clinical outcome	Complete resolution of symptoms and palatal lesion	Complete healing of palatal lesion with sustained clinical improvement	Sustained symptom resolution without clinical recurrence	Partial symptomatic improvement despite persistent radiological disease
Final status	Alive, clinically well, no recurrence during follow-up	Alive, clinically well, no recurrence during follow-up	Alive, disease-free at 4-year follow-up	Survived approximately 1 year after diagnosis; persistent radiological disease

This table reflects the overall clinical course of the patients.

**Table 3:** Safety summary.

Safety Parameter	Case 1	Case 2	Case 3	Case 4
Baseline clinical safety considerations	Diabetes mellitus and hypertension	Chronic kidney disease/chronic renal failure documented at baseline; solitary functioning kidney; treatment-resistant hypertension; impaired renal function	Diabetes mellitus; baseline impaired renal function	Diabetes mellitus and hypertension
Baseline serum creatinine (mg/dL)	1.05 13/01/2025	1.48 01/03/2024	1.74 16/05/2022	1.4 12/03/2025
Follow-up serum creatinine (mg/dL)	1.17 13/06/2026	3.3 03/12/2024	1.54 14/06/2026	Not available
Baseline liver function tests	Not available	Not available	Not available	Not available
Follow-up liver function tests	13/06/2026 SGPT: 41 U/L SGOT: 30 U/L Total bilirubin: 1.56 mg/dL	03/12/2024 SGPT: 22.75 U/L SGOT: 27 U/L Total bilirubin: 0.23 mg/dL	14/06/2026 SGPT: 17.7 U/L SGOT: 19.1 U/L Total bilirubin: 0.33 mg/dL	Not available
Clinical surveillance for adverse effects	Clinical monitoring during treatment and routine follow-up; no treatment-related adverse effects documented in available records	Clinical monitoring during treatment and routine follow-up; progressive renal dysfunction documented during follow-up	Clinical monitoring during treatment and routine follow-up; no treatment-related adverse effects documented in available records	Clinical monitoring during treatment and routine follow-up; no treatment-related adverse effects documented in available records
Fumigation-related observations	See overall observation below	See overall observation below	See overall observation below	See overall observation below
Serious or clinically significant adverse events documented	None documented as treatment-related in available records	Progressive renal dysfunction with increase in serum creatinine from 1.48 mg/dL to 3.3 mg/dL	None documented as treatment-related in available records	None documented as treatment-related in available records
Safety interpretation	Available follow-up renal and hepatic parameters did not demonstrate substantial deterioration. However, unavailable baseline liver function tests and non-standardised monitoring preclude definitive safety conclusions.	Serum creatinine increased from 1.48 to 3.3 mg/dL during follow-up, representing an important safety concern. Given the patient’s pre-existing chronic kidney disease and other renal risk factors, the cause cannot be determined; a treatment-related contribution cannot be completely excluded.	Serum creatinine decreased from 1.74 mg/dL to 1.54 mg/dL in the available follow-up measurement, and available follow-up liver parameters did not demonstrate substantial abnormalities. However, non-standardised monitoring and unavailable baseline liver function tests preclude definitive safety conclusions.	Laboratory safety monitoring was limited. Available clinical records did not document clinically significant treatment-related adverse effects; however, the absence of systematic laboratory monitoring precludes conclusions regarding treatment safety.
Overall safety assessment limitation	Retrospective, non-standardised, and incomplete monitoring; no predefined adverse-event assessment protocol; baseline liver function tests unavailable	Retrospective, non-standardised, and incomplete monitoring; no predefined adverse-event assessment protocol; baseline liver function tests unavailable; causality of renal deterioration cannot be determined	Retrospective, non-standardised, and incomplete monitoring; no predefined adverse-event assessment protocol; baseline liver function tests unavailable	Retrospective, non-standardised, and incomplete monitoring; no predefined adverse-event assessment protocol; limited laboratory follow-up

This table reflects the safety summary for the patients.

## Data Availability

This article presents a descriptive clinical case series derived from routine clinical care rather than a prospective protocol-based research study. The anonymised clinical data supporting the findings are presented within the article and its Supplementary Materials. Additional source data, derived from confidential patient medical records, are available to the Editorial Office or reviewers upon reasonable request for verification purposes. These records are not publicly available because unrestricted disclosure could compromise patient privacy and confidentiality and may be inconsistent with the informed consent provided by the patients and applicable legal and ethical requirements.

## Supplementary Materials

The following supporting information can be downloaded at: https://www.mdpi.com/article/10.3390/jof12100781/s1, Figure S1: Case 3 CT Report Before (A) and After (A) Ayurveda Treatment; Table S1: Details of Ayurveda formulations used; File S1: Clinical Course Timeline.
