# Supplementary material for: Clinical Course of Four Patients with Mucormycosis Managed Predominantly with Ayurveda-Based Interventions: A Case Series
Source: J Fungi (Basel). 2026 Oct 19;12(10):781. doi: 10.3390/jof12100781 (PMC13706485; doi:10.3390/jof12100781)
Supplement: Supplementary file 1 [file jof-12-00781-s001.zip › Table S1. Details of Ayurveda formulations used.pdf]

Intervention(Manufacture name with manufacturing licence number	Particulars	Case 1 (Duration in Days) And Dose	Case 2 (Duration in Days) And dose	Case 3 (Duration in Days) And Dose	Case 4 (Duration In days) And Dose	Reference and Rational of use
Brihat Vata Chintamani Rasa (Oral)(Shree Dhootpapeshwar Ltd., AYU-150)	Herbo mineral formulation containing purified Rasa (Purified Mercury), Gandhaka (Purified Sulfur), Abhraka Bhasma (Calcinated Mica)	150 (125 mg) 1 tab twice a day	120 (125 mg) 1 tab thrice a day	105 (125 mg) 1 tab twice a day	120 (125 mg) 1 tab twice a day	Book-Bhaishajya Ratnavali, 26/145-148, InFdicated in disease of Vata dosha (ITA-2.1.1.1 Vata, dosha regulating movement and cognition) and Rasayana (ITA-9.1.35 Rejuvenative therapy)
Krumi Kuthar Rasa (Oral) (Dhanvantari Guj.Herb, GA-428)	Herbo mineral Formulation containing Kajjali (Black Sulfide of Mercury), Tankana Bhasma (Borax)	120 (125 mg) 2 tab thrice a day	120 (125 mg) 2 tab thrice a day	105 (125 mg) 2 tab thrice a day	120 (125 mg) 2 tab thrice a day	Book - Ayurved sara Sangrah (Page no 323), Indicated in Krimi roga (Disease of Krimi, ITA-5.13.4 Internal worm Infestation)
Krumighna Vatika (Oral) (Nagarjuna herbal concentrates pvt. Ltd, 69/25-D/88)	Herbal Formulation containing Vidanga (Embelia ribes), Ajamoda (Apium graveolens), Haritaki (Terminalia chebula)	150 (250 mg) 2 tabs thrice a day	120 (250 mg) 2 tabs thrice a day	105 (250 mg) 2 tabs thrice a day	120 (250 mg) 2 tabs thrice a day	Book- Sahasrayogam Indicated in Krimi roga (Disease of Krimi, ITA-5.13.4 Internal worm Infestation)
Serankottai Nei Capsule (Intra nasal) (SKM siddha & Ayurveda company pvt. Ltd, 739)	Herbal combination containing Shuddha Bhallataka (Semecarpus anacardium), Milk, Ghee	120 1 capsule (10-15 ghee drops) twice a day as a nasal drop (Half a	120 1 capsule (10-15 ghee drops) thrice a day as a nasal drop (Half a	105 1 capsule (10-15 ghee drops) twice a day as a nasal drop (Half a	120 1 capsule (10-15 ghee drops) twice a day as a nasal drop (Half a	Book- Siddha Vaithya Thirattu Indicated in Kapha vyadhi (disease of Kapha, ITA-2.1.3.1 Kapha, dosha responsible for cohesiveness)

		capsule has to be instilled in both nostrils)	capsule has to be instilled in both nostrils)	capsule has to be instilled in both nostrils)	capsule has to be instille d in both nostrils)	
Boniheal Tablet<br>(Oral)<br>(AIMIL pharmaceuticals (INDIA) pvt. Ltd. – HP-15/Ay)	Contains mainly Godanti Bhasam (Classical Ayurvedic Preparation, Prcd.) –, Jahar Mohra Pishti (Classical Ayurvedic Preparation, Prcd.) –, Shalaki (Boswellia serrata, Exd.) –, Mukta Shukti Bhasam (Classical Ayurvedic Preparation, Prcd.) –, Sudh Laksha (Laccifera lacca purified, Secretion. Prcd.) –, Raal (Shorea robusta, Exd.) –, Vanshlochan (Bambusa sps., Sili. Concre.) –, Yashad Bhasam (Classical Ayurvedic Preparation, Prcd.) –, Shudh Shilajeet (Black bitumen purified, Prcd.) – ,etc.	150<br>250 mg 1 tab twice a day	120<br>250 mg 1 tab twice a day	105<br>250 mg 1 tab twice a day	120<br>250 mg 1 tab twice a day	Ayurveda Proprietary Medicine.<br>Indicated to strengthen the bony tissue
Vidanga Choorna<br>(Oral)<br>Single herb powder	Herbal Powder contains Vidanga (Embelia ribes)	90<br>(5–10 gm) To drink and eat food with vidang choorna	120<br>(5–10 gm) To drink and eat food with vidang choorna	105<br>(5–10 gm) To drink and eat food with vidang choorna	120<br>(5–10 gm) To drink and eat food with vidang choorn	Book- Bhava Prakash Indicated as the Best herb for Krimi (ITA-5.13.4 Internal worm Infestation)

		added into it	added into it	added into it	a added into it	
Palasha Choorna + Bhallataka Choorna + Vidanga Choorna<br>(Intra nasal fumigation)<br><br>Single herb Powder	Herbal Formulations contains powder of Palash (Butea monosperma), Bhallathaka (Semecarpus anacardium), Vidanga (Embelia ribes)	90<br>Mix all togethe r and the patient has to inhale and exhale the smoke generat ed in it mornin g and in the evening	120<br>Mix all togethe r and the patient has to inhale and exhale the smoke generat ed in it mornin g, afterno on and in the evening	105<br>Mix all togethe r and the patient has to inhale and exhale the smoke generat ed in it mornin g and in the evening	120<br>Mix all togethe r and the patient has to inhale and exhale the smoke generat ed in it mornin g and in the evening	Palash choorna Book- Bhava Prakash<br>Indicated as the Best herb for Krimi (ITA-5.13.4 Internal worm Infestation), Bhallatak choorna (Indicated in Kapha vyadhi disease of Kapha, ITA-2.1.3.1 Kapha, dosha responsible for cohesiveness)
Vyadhi Harana Rasa<br>(Oral)<br><br>(Krushan gopal Ayurved Bhavan – 539-D)	Herbo mineral formulation containing purified Rasa (Purified Mercury), Gandhaka (Purified Sulfur), Hartal (Purified Arsenic trisulfide, manshila (Purified Arsenic disulphide)	90<br><br>75 mg twice a day	120<br><br>75 mg thrice a day	105<br><br>75 mg twice a day	0	Rasa tantrasar and siddha prayoga-1-indicated in Nadivrana (ITA-5.56.1-Deep ulcer with fistula formation)
Panchtiktaka Kwatha<br>(Oral)<br>(Panchamrit herbals-GA/1762)	Herbal formulation containing mainly **Guduchi** *(Tinospora cordifolia),* **Nimba** (*Azadirachta indica),* **Vasa** (*Adhatoda vasica)*, **Kantakari** *(Solanum xanthocarpum),* **Patola** *(Trichosanthes dioica)*	90<br><br>15 ml twice a day	0	0	0	Book- Ras Tantra Saar & Siddha Prayog Sangrah Volume 1, Indicated to strengthen Asthi dhatu (ITA-2.2.10 Bone, The fifth of the seven fundamental structural components. Its function is to provide bodily stability)

Netra Sudarshana Arka (Eye Drops)<br>(Topical (Ophthalmic)<br>(Krushan gopal Ayurved Bhavan – 539-D)	Eye drop containing Palash (Butea monosperma)	150<br>2 eye drops in affected eye 3 times a day	120<br>2 eye drops in affected eye 3 times a day	0	120<br>2 eye drops in affected eye 3 times a day	Book- Ras Tantra Saar & Siddha Prayog Sangrah Volume 1, Indicated in netra roga (ITA-5.66.1 Eye disorders)
Panchtiktaka Guggulu Ghrita tablet<br>(Oral)<br>(Jamna herbal research Ltd-MP/25D/11/139)	Herbal formulation containing mainly **Guduchi** *(Tinospora cordifolia),* **Nimba** (*Azadirachta indica),* **Vasa** (*Adhatoda vasica)*, **Kantakari** *(Solanum xanthocarpum),* **Patola** *(Trichosanthes dioica)*	60<br>250 mg, 2 tab twice a day	0	0	120<br>250 mg, 2 tab twice a day	Book- Ras Tantra Saar & Siddha Prayog Sangrah Volume 1, Indicated to strengthen Asthi dhatu (ITA-2.2.10 Bone, The fifth of the seven fundamental structural components. Its function is to provide bodily stability)
Vranaphari Rasa<br>(Oral)<br>(Shri Jwala Ayurved Bhavan – 15/1977)	Herbo mineral formulation containing purified Rasa (Purified Mercury), Gandhaka (Purified Sulfur), Hartal (Purified Arsenic trisulfide, manshila (Purified Arsenic disulphide) and guggulu (Commiphora wightii)	60<br>(125 mg) 1 tab twice a day	120<br>(125 mg) 2 tab twice a day	0	120<br>(125 mg) 1 tab twice a day	Rasa tantrasar and siddha prayoga-2 indicated in Drusht vrana (ITA-5.54.7 Septic non healing ulcer)
